# Talar OsteoPeriostic grafting from the Iliac Crest (TOPIC) for lateral osteochondral lesions of the talus: operative technique

**DOI:** 10.1007/s00064-022-00789-0

**Published:** 2023-01-09

**Authors:** Jari Dahmen, Quinten G. H. Rikken, Gino M. M. J. Kerkhoffs, Sjoerd A. S. Stufkens

**Affiliations:** 1 Department of Orthopedic Surgery and Sports Medicine, Amsterdam Movement Sciences, Amsterdam UMC, Location AMC, University of Amsterdam, Meibergdreef 9, 1105 AZ Amsterdam, The Netherlands; 2 Academic Center for Evidence based Sports medicine (ACES), Amsterdam UMC, Amsterdam, The Netherlands; 3 Amsterdam Collaboration for Health and Safety in Sports (ACHSS), International Olympic Committee (IOC) Research Center, Amsterdam UMC, Amsterdam, The Netherlands; 4 Department of Orthopedic Surgery, University of Amsterdam, Meibergdreef 9, 1105 AZ Amsterdam, The Netherlands

**Keywords:** Ankle, Cartilage regeneration, Osteochondral autograft, Iliac crest, Transplantation technique, Talar osteochondral defect, TOPIC, Sprunggelenk, Knorpelregeneration, Osteochondrales autologes Transplantat, Beckenkamm, Transplantationstechnik, Osteochondraldefekt des Talus, TOPIC

## Abstract

**Objective:**

To provide a natural scaffold, good quality cells, and growth factors to facilitate replacement of the complete osteochondral unit with matching talar curvature for large osteochondral lesions of the lateral talar dome.

**Indications:**

Symptomatic primary and non-primary lateral osteochondral lesions of the talus not responding to conservative treatment. The anterior–posterior or medial–lateral diameter should exceed 10 mm on computed tomography (CT) for primary lesions; for secondary lesions, there are no size limitations.

**Contraindications:**

Tibiotalar osteoarthritis grade III, malignancy, active infectious ankle joint pathology, and hemophilic or other diffuse arthropathy.

**Surgical technique:**

Anterolateral arthrotomy is performed after which the Anterior TaloFibular Ligament (ATFL) is disinserted from the fibula. Additional exposure is achieved by placing a Hintermann distractor subluxating the talus ventrally. Thereafter, the osteochondral lesion is excised in toto from the talar dome. The recipient site is micro-drilled in order to disrupt subchondral bone vessels. Thereafter, the autograft is harvested from the ipsilateral iliac crest with an oscillating saw, after which the graft is adjusted to an exactly fitting shape to match the extracted lateral osteochondral defect and the talar morphology as well as curvature. The graft is implanted with a press-fit technique after which the ATFL is re-inserted followed by potential augmentation with an InternalBrace™ (Arthrex, Naples, FL, USA).

**Postoperative management:**

Non-weightbearing cast for 6 weeks, followed by another 6 weeks with a walking boot. After 12 weeks, a computed tomography (CT) scan is performed to assess consolidation of the inserted autograft. The patient is referred to a physiotherapist.

## Abstract (de)

**Operationsziel:**

Ziel der Behandlung ist es, große primäre und sekundäre laterale Defekte in der Talusrolle mit einem autologen osteochondralen Transplantat zu füllen. Damit wird das natürliche Knochengerüst wieder aufgebaut, werden qualitativ hochwertige Zellen und Wachstumsfaktoren bereitgestellt, und auch die Kontur der Talusrolle wird wiederhergestellt.

**Indikationen:**

Symptomatische primäre und sekundäre Osteochondraldefekte der lateralen Talusrolle nach fehlgeschlagener Physiotherapie. Größere Läsionen (anteroposteriorer oder mediolateraler Durchmesser > 10 mm in der Computertomographie) bei primären Läsionen – für sekundäre Läsionen gibt es keine Einschränkungen.

**Kontraindikationen:**

Generalisierte Osteoarthrose im gesamten Sprunggelenk, aktive Infektion, Malignität, Hämophilie oder andere diffuse Arthropathien.

**Operationstechnik:**

Eine anterolaterale Arthrotomie wird durchgeführt, danach das Ligamentum talofibulare anterius (ATFL) am fibulären Ursprung abgelöst. Zusätzliche chirurgische Exposition kann durch das Anbringen eines Hintermann-Distraktors erreicht werden, danach wird der Talus nach ventral subluxiert. Zunächst wird der osteochondrale Defekt vollständig aus der Talusrolle herausgeschnitten. Die Empfängerstelle wird mit Mikrobohrungen versehen, um die subchondralen Knochengefäße zu öffnen. Anschließend wird das autologe Knochentransplantat mit einer oszillierenden Säge am ipsilateralen Beckenkamm entnommen, wonach das Transplantat auf eine genau passende Form gebracht wird, die dem entfernten osteochondralen Defekt und der Morphologie und Krümmung des Talus entspricht. Das Implantat wird mit einer Press-Fit-Technik implantiert. Danach wird das ATFL an der Fibula reinseriert, bei Bedarf mit einer zusätzlichen Augmentation mit einem InternalBrace™ (Arthrex, Naples, FL, USA).

**Nachbehandlung:**

Unbelastete Mobilisation im Unterschenkelgips für 6 Wochen, vollbelastete Mobilisation in einer Unterschenkelorthese (Walker) für weitere 6 Wochen. Nach 12 Wochen: Computertomographie zur Analyse der Konsolidierung/Integration des Transplantats. Verordnung von Physiotherapie.

## Introductory remarks

An osteochondral lesion of the talus (OLTs) is characterized by damage to the talar articular cartilage and the underlying subchondral bone. These lesions are predominantly caused by ankle fractures or ankle sprains, but can also have an idiopathic or genetic origin [11, 33]. This is evidenced by recent literature from which we know that 45% of acute ankle fractures are associated with concomitant damage to the (osteo)chondral unit of the ankle, with the talar bone being the most frequently reported location [22].

Currently, there is no superior treatment for primary or secondary OLTs [9, 18]. However, it is recommended to initiate treatment of patients with painful OLT with an appropriate conservative treatment protocol in the context of individualized care. This treatment protocol can consist of supervised neglect, a period of immobilization, (multiple) injections of hyaluronic acid, supervised physiotherapy, and specific prescription of insoles [9, 34, 42]. However, the majority of patients will remain symptomatic and progress to a surgical strategy. The choice of the specific surgical intervention in question largely depends on specific lesion factors, such as the primary or non-primary nature of the lesion, lesion dimensions, and lesion morphology [31].

For small primary defects, first-line surgical management may consist of (arthroscopic) bone marrow stimulation or retrograde drilling [7, 9, 18]. For lesions that are amenable to fixation (fragmentous lesions), arthroscopic and open internal fixation procedures have been proven to be effective [15, 16, 19, 29, 30]. In the case of relatively large (cystic) lesions of the talar dome or in the case of lesions that have failed prior surgical intervention(s), it is probable that more invasive surgical interventions will be necessary in order to adequately address the patients’ complaints [18]. These surgical interventions may consist of transplantation techniques such as (osteo)chondral transplantation, including the classical osteochondral autograft transfer system (OATS) and chondrocyte implantation techniques [38]. Alternatively, one can consider bulk allografting—a surgical technique used whenever the lesion can be considered “massive” and a surgeon expects that insufficient autografting material will be obtained. The aforementioned techniques show adequate clinical efficacy in terms of clinical success rates, as previously assessed by Dahmen et al. [9] and Lambers et al. [18]. However, it can be stated that each of the surgical techniques for larger and/or cystic OLTs are associated with specific downsides. For example, it has been proven that the OATS procedure can cause a donor-site morbidity incidence rate ranging from 11% to 35% when the graft is harvested from the ipsilateral femoral condyle [9, 12]. A different example of a specific downside can be nonunion, whereas for the cartilage implantation techniques, the financial investment can be regarded as a downside [1].

A potential alternative to the aforementioned techniques may be harvesting of autologous osteoperiosteal grafts. Cylindrical use of osteoperiosteal grafts has previously been documented by Hu et al. [12] and Chen et al. [6], and yielded clinically effective outcomes. It is, however, a common downside of cylindrical grafts that the size of the cylindrical autograft is static and determined preoperatively [39].

In order to overcome the aforementioned downsides, a novel technique for the treatment of large talar osteochondral lesions has recently been developed and was previously described in the present journal for medially located lesions [14]. The Talar OsteoPeriostic grafting from the Iliac Crest (TOPIC) procedure uses the ipsilateral iliac crest with the overlying periosteal layer as the harvesting location for the autograft. The periosteal layer has the potential to induce articular cartilage-like tissue regeneration as the cambium layer of the periosteum contains chondrocyte precursor cells [13, 23, 26]. The TOPIC bone–periosteal transplant meets the three requirements for tissue engineering for bone and cartilage repair: a source of cells, a scaffold, and local growth factors [3, 21, 32, 36]. In addition, the curvature of the iliac crest is highly similar to the curvature of the talar bone, and the harvested graft can be exactly fitted to the excised OLT, leading to a press-fit graft [25].

However, the previously published description of the TOPIC technique was limited to medially located osteochondral lesions of the talus. We know from a recent publication by van Diepen et al. [40] that 24% of the lesions that are located on the talar bone are specifically located on the lateral talar dome. The previous description focusing on medially located lesions can be considered substantially different to lateral lesions, as for the medially located lesions, a distal tibial osteotomy is considered a necessary first step in the procedure through which appropriate visualization is created. Laterally located lesions can be approached without the use of distal tibial osteotomies, though require different steps for approaching the lesion. This substantiates the necessity of a detailed presentation of the surgical technique of the lateral TOPIC procedure consisting of a specific stepwise approach.

The purpose of the current study is therefore to describe the surgical technique of the newly developed lateral TOPIC procedure, to evaluate potential pearls and pitfalls, and to present the first clinical outcomes of patients who have undergone the lateral TOPIC procedure.

## Surgical principle and objective

Large symptomatic osteochondral lesions to the talus of primary or non-primary nature are challenging. Currently, there are many surgical techniques available for this indication, with their individual advantages and disadvantages. Over the past decades, surgical and scientific attention has been focused on medially located lesions of the talar dome. However, as we are aware of the fact that 24% of the OLTs are located on the lateral talar dome and that the laterally located OLTs are associated with chronic lateral ankle instability, there is a clinical need to present surgical techniques that are amenable and effective for laterally located OLTs [2, 37, 40, 41, 43]. Moreover, it is stated that concomitant lateral ankle instability is considered a negative prognostic factor regarding the clinical outcomes of surgical treatment of OLTs [17, 20, 27]. Consequently, this highlights the clinical relevance and importance of addressing laterally located lesions appropriately in the case of lesions of large (cystic) nature and in case of concomitant (chronic) lateral ankle instability. A novel innovative surgical technique for the surgical treatment of large OLTs has recently been developed and was previously described in the present journal. However, this technique focused exclusively on medially located OLTs, not on laterally located OLTs with possible concomitant (chronic) lateral ankle instability [14]. The present surgical technique paper therefore describes the surgical technique of treatment of large (cystic) primary and secondary lateral osteochondral lesions of the talus. The technique comprises (antero)lateral arthrotomy, press-fit talar autograft transplantation from the ipsilateral iliac crest, and lateral ankle ligament reconstruction potentially augmented with an InternalBrace™ (Arthrex, Naples, FL, USA). The technique provides a natural scaffold, good quality cells, and growth factors, facilitating replacement of the complete osteochondral unit—a triad considered to be of vital importance in the treatment of these defects. Moreover, the technique can directly and simultaneously address (chronic) lateral ankle instability, which is a common concomitant diagnosis in painful osteochondral lesions of the talus [2, 37, 40, 41, 43].

## Advantages

- Concomitant lateral ankle ligament repair/reconstruction (with potential augmentation by InternalBrace™) in the case of (chronic) lateral ankle instability (instead of ligament reconstruction)
- Similar surface geometry and curvature of the talar dome compared to the iliac crest [25].
- Iliac crest graft provides scaffold, cells, and growth factors [3, 21, 32, 36].
- No need for an osteotomy due to the location of the defect (whenever the lesion is located on the anterior 80% of the lateral talar dome; if the lesion is located on the posterior 20% of the lateral talar dome, a fibular osteotomy can be considered).
- Cost effective.
- Single-stage technique.
- Limited operation time.

## Disadvantages

- Complications specifically for the harvest site, the iliac crest, and hyp(er)aesthesia skin [10].
- Potential complications of the superficial peroneal nerve due to the surgical approach.
- Secondary osteoarthritis.

## Indications

- Symptomatic large (cystic) laterally located osteochondral lesion of the talar dome with deep ankle pain not responding to conservative treatment with or without the presence of (chronic) lateral ankle instability;
  - anterior-posterior or medial-lateral lesion diameter should exceed 10 mm on computed tomography (CT),
  - the depth of the defect is no limitation for this procedure.
- Both primary and secondary lesions are eligible for treatment with TOPIC
- In case of malalignment, a corrective osteotomy can be considered.

## Contraindications

- Tibiotalar osteoarthritis grade III (Kellgren and Lawrence).
- Malignancy.
- Active infectious ankle joint pathology.
- Hemophilic or other diffuse arthropathy.

## Patient information

- Surgical risks include infection, hematoma, thromboembolic event, wound healing problems.
- Transient or permanent nerve damage leading to hyper/o aesthesia of the superficial peroneal nerve.
- Non-weightbearing cast for 6 weeks, followed by a walking boot for another 6 weeks.

## Preoperative workup

At the Department of Orthopedic Surgery and Sports Medicine of the Amsterdam University Medical Centers (AUMC) we have a standardized workup for all patients: patients are carefully screened for patient history (including recurring ankle sprains and/or a feeling of giving way of the ankle) and undergo a thorough physical examination of both lower extremities and ankles, during which specific attention is paid to recognizable pain on palpation over the site of the osteochondral lesion(s) with the ankle in plantar flexion [31]. Special emphasis is also put on the range of motion of the ankle joint, especially plantar flexion [8]. Moreover, a highly important part of the physical examination is to carefully assess whether there is also presence of (chronic) lateral ankle instability of symptomatic nature.

For confirmation of the diagnosis and as a preoperative sizing and planning tool, an additional dual-energy computed tomography scan (CT) is made. At our center, experienced fellowship-trained musculoskeletal radiologists and the orthopedic surgeons assess all OLTs for lesion size, morphology, shaping, and curvature. Lesion size is assessed three-dimensionally, taking into account the morphology of the lesion in a simultaneous fashion in order to yield a three-dimensional visualization and representation of the lesion allowing for a personalized approach [31]. The three-dimensional analysis concerns measuring the sclerotic borders of the lesion. This is performed by measuring the lesion from the most anterior point to the most posterior point (AP direction), as well as from the most medial point to the most lateral point (ML direction), and finally, the depth is measured by measuring the distance from the joint line to the most distal part of a cyst most often including the caudal sclerotic zone of the cyst, as this will be removed accordingly. After careful shared decision-making process together with the patient and their family, the patient and the treating team will decide for the lateral TOPIC procedure together.

## Instruments and implants

- Coagulation knife
- Hohmann retractors
- Oscillating saw and/or chisel with thin blades
- Beaver knife
- Hemostatic gelatin sponge (Spongostan®; Ethicon, Somerville, NJ, USA)
- Impactor
- Standard orthopedic set
- Chisel set
- Hintermann distractor
- Ligament bracing equipment (InternalBrace™)

## Anesthesia and positioning

- General or spinal anesthesia
- Antibiotic prophylaxis (Cefazolin 2 g intravenous injection) is administered to each patient
- Supine position with a tourniquet applied around the thigh

## Surgical technique

**Fig. 1:**
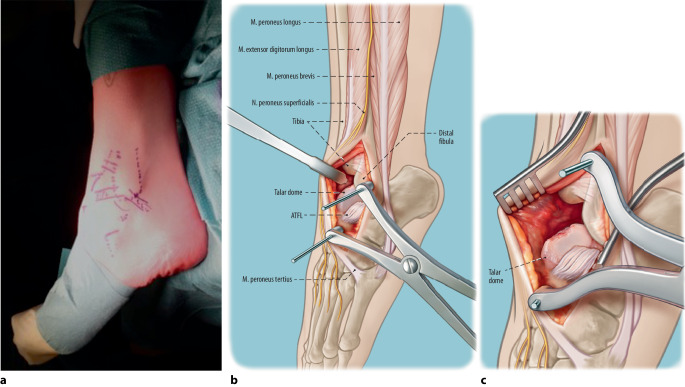
Anterolateral approach. After prepping and draping, a curvilinear incision of approximately 10 cm is made just anteriorly of the distal fibula, extending inferiorly towards the sinus tarsi joint (**a**). The anterolateral joint capsule is exposed as well as the inferior extensor retinaculum without damaging the superficial peroneal nerve. Hohmann retractors are placed in order to protect the neurovascular structures and the tendons. The anterior talofibular ligament (ATFL) and the anterior syndesmosis are exposed and identified. Potential anterolateral and/or anterocentral osteophytes are removed to increase exposure to the osteochondral lesion on the lateral dome. The ATFL is released from the footprint at the fibula, after which the Hintermann distractor is placed on the talar bone and the distal fibula (**b**). The talar osteochondral lesion is fully exposed due to anterior translation and subluxation of the talar dome (**c**). An alternative evidence-based approach is a fibular osteotomy, through which adequate exposure of the joint is also possible and could be performed based on the surgeon’s experience and preference or when the lesion is located on the far posterior part of the lateral dome (posterior 20%; [24])

**Fig. 2:**
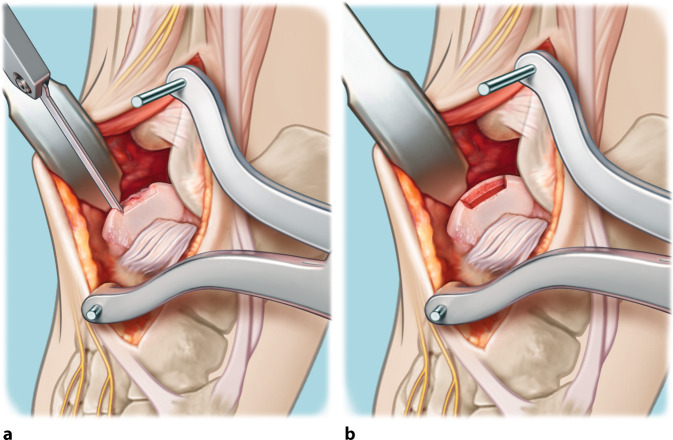
Excision of the diseased osteochondral defect. **a** After adequate exposure and identification of the lesion, the cartilage is incised in a rectangular fashion to preserve as much healthy cartilage as possible and to prevent the cartilage from rupturing in the following steps of the procedure. Potentially available cysts and/or present necrotic bone is resected en bloc using a chisel with thin blades and/or an oscillating saw. **b** End result: total removal of the premeasured osteochondral lesion of the talus, deep enough so that healthy bone can be observed

**Fig. 3:**
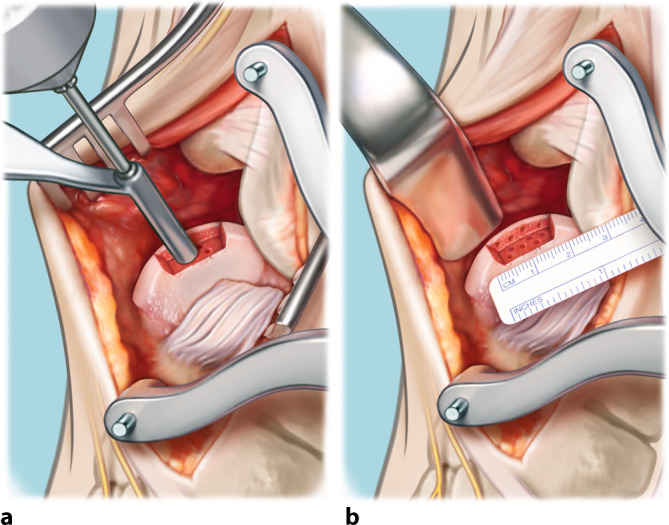
Drilling of the recipient’s bone base. The base of the autograft recipient is (micro-)drilled so that bone marrow stimulation by means of a 2.0-mm drill takes place (**a**). This step results in a totally debrided and premeasured (three dimensionally; anteroposterior, mediolateral, and depth) prepared recipient site with healthy bone present at the lesion’s base (**b**). In the case of the presence of cysts extending beyond the en bloc resection of the talar osteochondral lesion (OCL), additional debridement with curettage is performed, and these cysts will be filled with spongious bone coming from the iliac crest (Figs. 4 and 5)

**Fig. 4:**
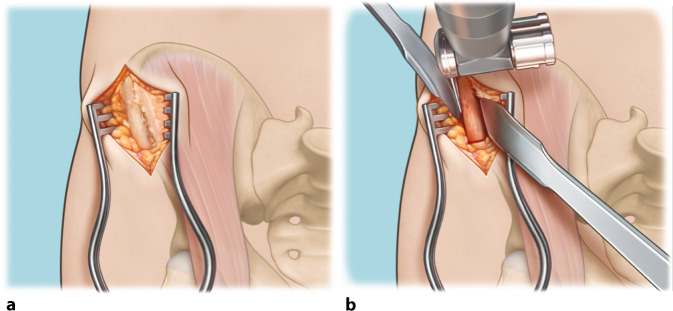
Measuring and harvesting the donor autograft from the iliac crest*. *Harvesting of the graft is initiated, keeping in mind the measurements of the excised osteochondral lesion of the talus (as illustrated in step 3) and combining these measurements with the predefined CT measurements. This is performed in order to correctly approximate the donor autograft. The goal is to oversize in all directions (anteroposterior and mediolateral diameter as well as depth) by 1 mm. The ipsilateral iliac crest is approached through a horizontal incision of 3.5 cm (**a**). The predefined measurements are applied three-dimensionally when harvesting the graft. The coagulation knife should not be used, in order to keep the periosteum attached to the bone. Subsequently, a monocortical, bicortical, or tricortical osteoperiosteal autograft from the ipsilateral iliac crest is harvested with the use of an oscillating saw and chisel (**b**). The combination of the width of the iliac crest and the graft size needed dictates the selection of a monocortical, bicortical, or tricortical graft. A hemostatic gelatin sponge (Spongostan®; Ethicon, Somerville, NJ, USA) is left in the iliac crest after harvesting

**Fig. 5:**
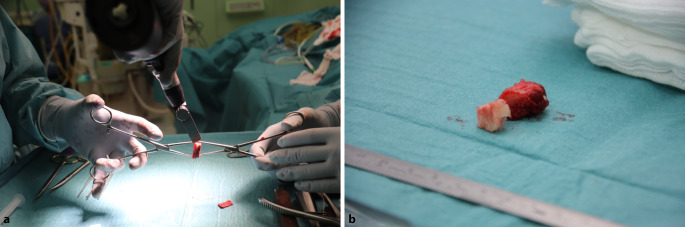
Adjusting the fitting shape of the autograft. The autograft is adjusted and fine-tuned with an oscillating saw to fit size, curvature, depth, and morphology of the excised osteochondral lesion of the talus (OLT), as described in our earlier publication ([14]; **a**). The graft should not be positioned too proud; it is best to place the graft 1 to 2 mm under the cartilage level of the talar dome in order to prevent a proud position of the graft. The graft will be placed press fit; 1 mm should be added to all the dimensions of the excised OLT. An additional trick is to compare the graft (right side of this image) to the excised OLT (in toto) in order to enhance similarity of the autograft to the excised OLT (**b**)

**Fig. 6:**
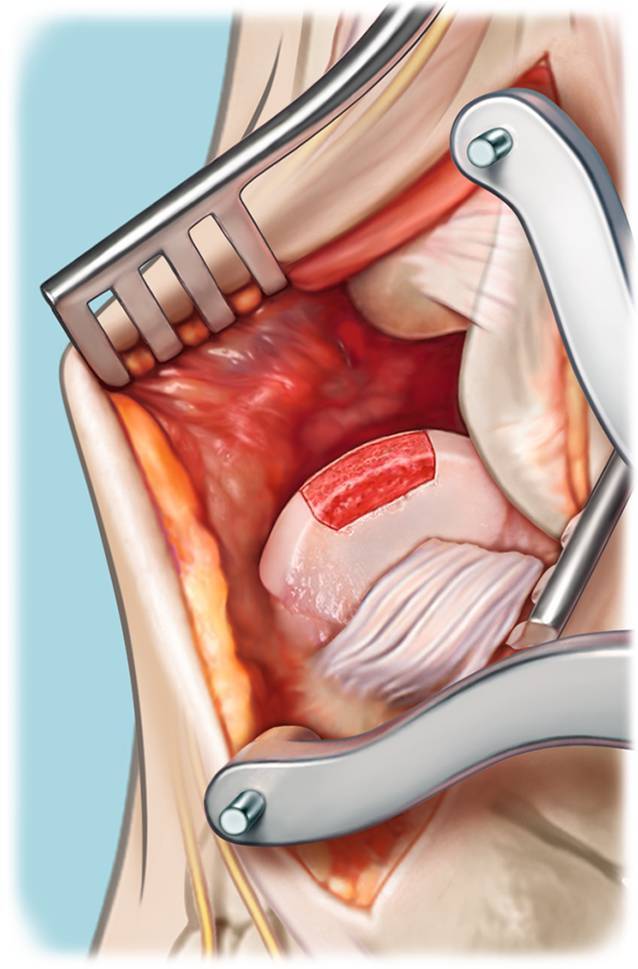
Implanting the graft through a press-fit technique. After trying and fitting the graft, the optimal fitting shape may be reached, after which the autograft is placed into the recipient site. Using a rounded impactor, the adjusted iliac crest autograft will be placed 1–2 mm underneath the level of the cartilage of the remaining talar dome. No screws are necessary to fixate the inserted autograft due to the press-fit nature of the present surgical technique. If there is instability of the graft, a potential alternative is to use one or two bioscrews to fixate the graft

**Fig. 7:**
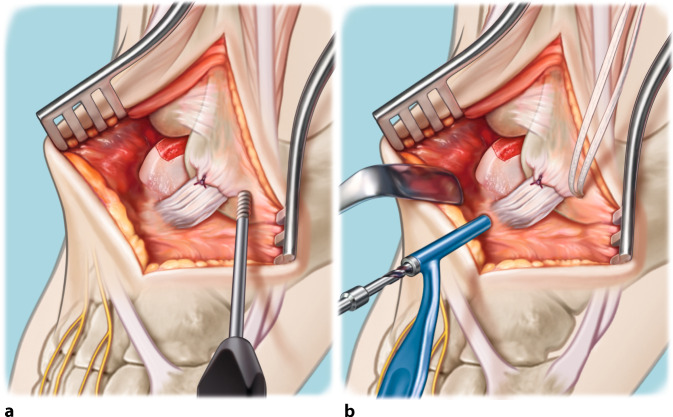
Augmentation with InternalBrace™. After inserting the autograft in a press-fit manner, the range of motion (ROM) of the ankle is properly analyzed. The footprint of the anterior talofibular ligament (ATFL) is cleared and cleaned, followed by reinsertion of the ATFL with use of a bone anchor 2.4–3.5 mm and the overlying periosteum. In the case of a fragile ATFL, augmentation of the ATFL re-insertion with an InternalBrace™ technique is performed with positioning of anchors on the distal fibula and the nonarticulating surface of the lateral border of the talar dome (**a,b**). Both the final ROM and the lateral ankle stability are tested, after which the incision layers are closed and additional rinsing takes place

(Figs. 1, 2, 3, 4, 5, 6, 7)

## Special surgical considerations

- A preoperative dual-energy CT scan allows for optimal radiological assessment of lesion morphology and lesion size. This method yields a 3D visualization and representation of the lesion, allowing for a personalized approach both preoperatively as well as intraoperatively [31].
- A concomitant lateral ankle ligament repair/reconstruction with the potential augmentation with an InternalBrace™ is clinically relevant and surgically located in an optimal position due to the (antero)lateral approach, allowing for prognostically positive outcomes [17, 20, 27].
- The iliac crest autograft transplantation is adjusted for and customized for each individual patient.
- Press-fit autograft transplantation allows for a one-stage procedure and quick rehabilitation, without the clinical necessity of intra-articular screw removal.
- An optimal (osteoperiosteal) graft to replace an osteochondral unit has the following characteristics: natural scaffold, good quality cells, and growth factors [28, 35].

## Postoperative management

Postoperatively a lower leg splint is provided for the first 24 h, after which a fresh circular non-weightbearing lower leg cast is applied for 2 weeks. 2 weeks postoperatively, the stitches are removed and the patient is motivated and allowed to perform dorsoplantar motion of the ankle so that postoperative deficits in range of motion in the dorsoplantar direction can be addressed. Moreover, these movements may simulate the progenitor cells from the periosteum to produce an optimal chondral layer following stimulation through ankle joint motion [4, 5].

The non-weightbearing period is 6 weeks and at the 6‑week follow-up, a walking boot is applied for another 6 weeks, allowing the patient (gradual) weightbearing as tolerated. At 12 weeks postoperatively, a dual-energy CT scan is performed in order to assess consolidation of the inserted iliac crest autograft. At this particular outpatient visit, a personalized physiotherapeutic plan is provided to the patient and the physiotherapist in order to allow for evidence-based individualized build-up of activities, with gradual and protocol-based return to sport and work. In order to monitor progress, the patient is clinically assessed at 6 months and 1 and 2 years postoperatively. The patient is advised to not perform any axial-loading peak forces for at least 6 months postoperatively.

## Errors, hazards, and complications

**Fig. 8:**
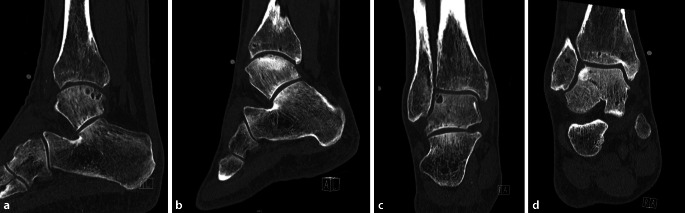
Preoperative and postoperative (1-year follow-up) CT scans of the same patient. **a** Sagittal preoperative CT scan, **b** sagittal postoperative CT scan, **c** coronal preoperative CT scan, **d** coronal postoperative CTscan

At every follow-up, patients are checked for potential complications, such as crest pain, hyp(er)aesthesia in the crest and ankle joint region, infections, ankle joint synovitis, and postoperative pain. A CT scan will be made at 3, 12, and 24 months after surgery to assess consolidation, talar remodeling, and ingrowth of the transplant (Fig. 8). Potential pitfalls:

- In case of inadequate restoration of lateral ankle biomechanics by means of lateral ankle ligament repair/reconstruction with potential augmentation with an InternalBrace™, persistent (symptomatic) lateral ankle instability may occur.
- Avulsion or fracture of the iliac crest when harvesting the graft too close to the anterior superior iliac spine.
- Proudness of the autograft might result in chondral wear or a kissing lesion to the opposing distal anterolateral corner of the ankle joint (fibula/distal tibia).
- Graft failure (rare).
- Ankle joint stiffness in dorsoplantar direction.
- Persistent ankle pain because of progressive osteoarthritis (OA) complaints.
- Potential osseous impingement of the lateral gutter in the case that the graft has slight overhang or in case of the presence of a lateral osteophyte or multiple osteophytes on the lateral side of the ankle joint.

## Declarations

For this article no studies with human participants or animals were performed by any of the authors. All studies mentioned in the article were in accordance with the ethical standards indicated in each case.
